# Multi-level Percutaneous Vertebroplasty for Multiple Spinal Metastases With Asymptomatic Epidural Compression: A Case-Based Example of Minimally Invasive Patient Management

**DOI:** 10.7759/cureus.72102

**Published:** 2024-10-22

**Authors:** Vladimir S Prandzhev, Donika I Vezirska

**Affiliations:** 1 Department of Neurosurgery, Military Medical Academy, Sofia, BGR

**Keywords:** breast cancer, breast cancer metastases, metastatic disease, minimally invasive metastasis management, percutaneous vertebroplasty, spinal metastases

## Abstract

Percutaneous vertebroplasty (PVP) is a minimally invasive procedure that allows for treating or preventing vertebral fractures resulting from trauma, osteoporosis, or oncological conditions. Metastatic spinal disease is a condition that necessitates mostly palliative care and pain management with minimal invasiveness. It could present with axial or localized back pain and may be associated with neurological deficit if compression of the spinal cord and/or the nerve roots is involved. The treatment of such patients may present a challenge and is usually accomplished with open surgery and instrumentation. However, the potential perioperative complications of invasive surgical procedures may significantly worsen the quality of life in patients with an already poor prognosis.

This case report presents minimally invasive management of multiple spinal metastases of the lumbar region in a 65-year-old female patient. She had a history of breast cancer and presented only with axial pain in the lumbar region and no neurological deficit. We performed a biopsy at the L3 level and multi-level PVP at the Th12-L5 levels; her pain diminished significantly, and she was mobilized later on the day of the surgery with a lumbar orthosis brace. Postoperative radiographic evaluation showed satisfactory cement distribution. The patient was subsequently referred to radiotherapy according to the decision of the multidisciplinary oncological committee.

## Introduction

Multiple spinal metastases are commonly regarded as part of the last and incurable stage of an oncological disease [1]. Symptoms may vary from a normal neurological status with tolerable localized back pain only to severe paraparesis with loss of bladder and bowel control [2,3]. The main indications for surgical treatment include pain, neurological deficits, and spinal instability. The decision to operate is objectively made according to the Spinal Instability Neoplastic Scale (SINS) scale [4]. 

However, major spinal surgery has a high risk for postoperative complications in patients with multiple spinal metastases [5]. This necessitates a complex multidisciplinary approach in such cases in order not to compromise the patient’s quality of life and to be as minimally intrusive as possible [6].

We present a case report demonstrating a minimally invasive management approach to a patient with multiple spinal metastases with no neurological deficit besides axial pain in the lumbar region.

## Case presentation

A 69-year-old woman was admitted to our clinic with intense pain in the lumbar region. Her medical history revealed partial resection of the right breast for breast cancer 20 years ago, and she had severe postoperative lymphedema of the right upper limbs. She had no motor or sensory deficit and no loss of bowel or bladder control (grade E according to the Frankel classification for spinal cord function).

**Figure 1:**
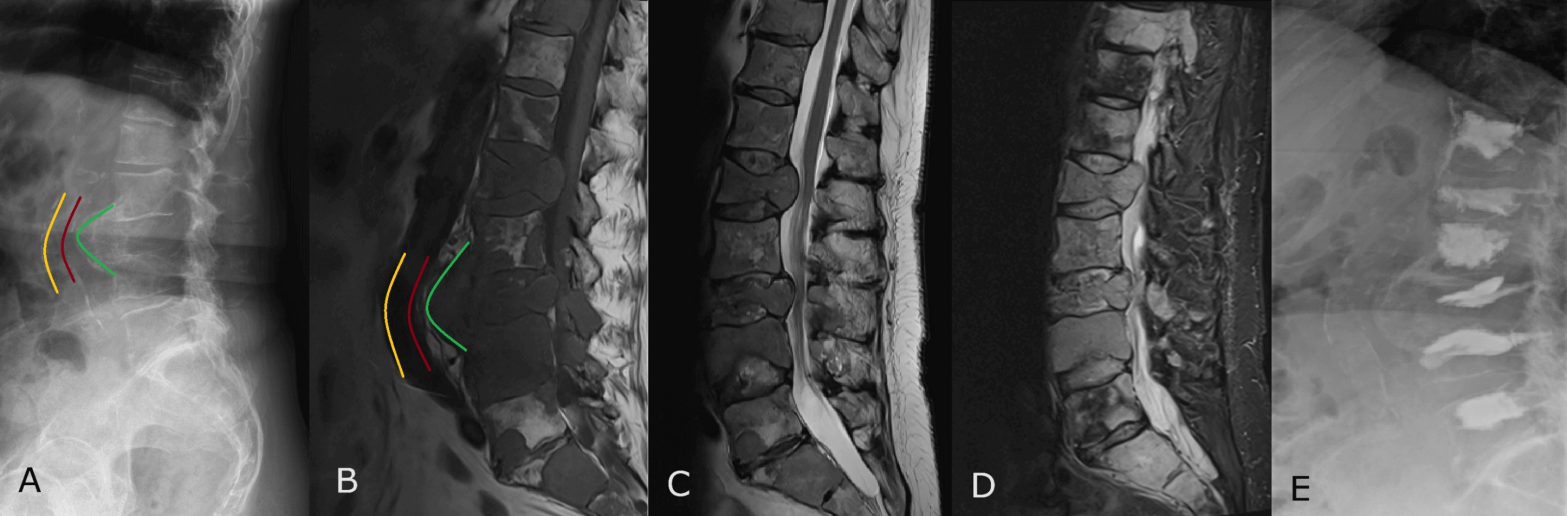
Pre- and postoperative images of multiple spinal metastases A) Preoperative radiographic evaluation showed an osteolytic lesion in the L3 vertebral body that compressed the large vessels anterior to it (the yellow line outlines the anterior wall of the large vessel, the red line outlines the posterior wall of the large vessel, and the green line outlines the anterior margin of the osteolytic lesion); B) T1-weighted MRI sagittal sequence correlated with the X-ray image (see the legend above); C) T2-weighted MRI sagittal sequence demonstrated the precise contours of the vertebral bodies; D) short tau inversion recovery (STIR) MRI sagittal sequence showing multiple vertebral metastases; E) Radiographic evaluation at postoperative day seven showed the distribution of bone cement with no extrusion towards the spinal canal

Radiographic examination revealed an osteolytic lesion of the L3 vertebral body, which seemed to be displacing the descending aorta and the inferior vena cava (Figure 1A). Further radiological examination by an MRI scan revealed multiple spinal metastases in the lumbar region with the destruction of multiple spinous processes and laminar arches along with the prominence of the tumor masses toward the spinal canal (Figures 1B-1D). T1-weighted images showed that the tumor mass at the L3 vertebral body did not engage the vascular structures anterior to it.

A percutaneous transpedicular biopsy was taken from the L3 vertebral body, and percutaneous vertebroplasty (PVP) was performed on the Th12-L5 levels. Our operative time clocked in at 46 minutes, and our blood loss was approximately 30 mL.

The postoperative period was uneventful. The patient was mobilized independently three hours after the operation and ambulated spontaneously. She had no additional neurological deficit (Frankel grade E). Her pain score went down from a Visual Analog Scale (VAS) score of eight preoperatively to two postoperatively. A lumbar orthosis brace was used in the early postoperative period.

A follow-up radiological examination was conducted on postoperative day seven. No cement extravasation towards the spinal canal was present, and there was no further compromise of the vertebrae (Figure 1E).

**Figure 2:**
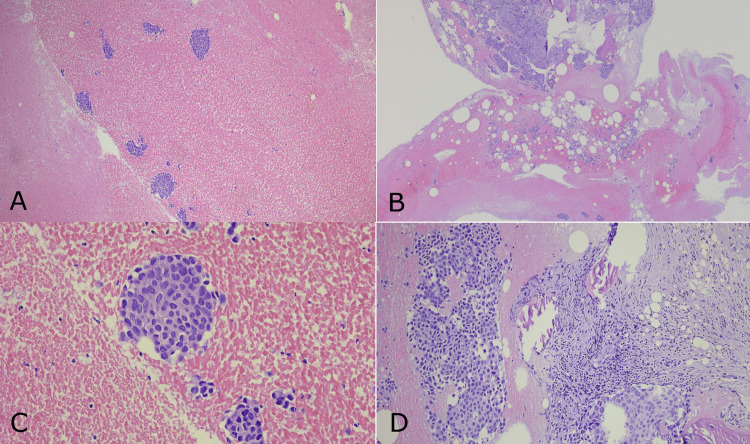
Hematoxylin and eosin (H&E) staining A) 2.5x magnification; B) 5x magnification; C-D) 10x magnification

Pathological examination confirmed metastatic disease from poorly differentiated invasive ductal breast carcinoma (Figures 2A-2D).

**Figure 3:**
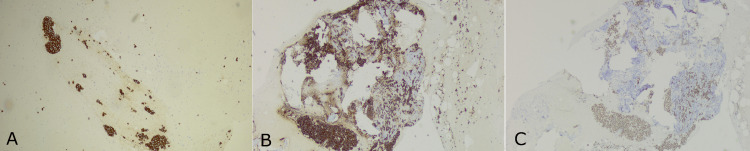
Immunohistochemical staining demonstrated positive breast cancer markers A-B) MCK staining; C) GATA staining

MCK and GATA3, as well as sporadic mammaglobin cell expression, were observed (Figures 3A-3C).

The receptor status was confirmed to be: ER - IS=3, PS=5, TS=8; PR negative; HER-2: 1+ (weakly positive incomplete membrane reaction in approximately 10% of the cells - HER-2-low); Ki67: ~ 15%. The exact histological type was determined as luminal type B.

The multidisciplinary oncological committee referred the patient for radiotherapy, target therapy, chemotherapy, and palliative care.

The patient came for a follow-up examination one month later. She was wearing her orthotic brace and taking 75 mg of pregabalin twice a day. She reported VAS 0 to one depending on the physical exertion during a certain day. No additional neurological deficit was present.

## Discussion

Currently, there are no clear guidelines concerning the treatment of multiple spinal metastases. Epidural spinal cord compression is one of the main factors to be considered to decide the precise treatment for the patient. According to the National Institute for Health and Care Excellence (NICE) guidelines on radiotherapy for spinal metastases, the committee recommends radiotherapy for asymptomatic spinal metastases with radiological signs of impending cord compression by an epidural tumor. This may prevent the appearance of a neurological deficit, although the evidence is not sufficient to recommend it routinely. Surgery to prevent further deformation and fracture of the vertebrae combined with radiotherapy shows better results than radiotherapy alone [7].

When it comes to surgery, the presence of neurological deficits and the improvement with conservative therapy are crucial to the decision. Percutaneous vertebroplasty can be successfully used in the management of spinal metastases to prevent burst fractures that occur under a significantly lower amount of axial load than those in a healthy vertebra. Common notions to keep in mind include the integrity of the posterior wall of the vertebral body, epidural spinal cord compression, and the deformation of the dural sac; they increase the risk of cement leakage towards the spinal canal, which may lead to neurological deficit [8]. A study by Jakobs et al. notes that cement volume and the asymmetrical distribution are not the main factors for stabilization, as 20% filling of the vertebra is not necessary to be exceeded [9].

Some studies showcase the use of tumor ablation (radiofrequency ablation (RFA) and microwave ablation (MWA)) before the injection of bone cement into the vertebral body [9-10]. This may help in achieving better local tumor control in addition to the prevention of fractures and the analgesic effect caused by the thermal reaction of the bone cement. Radiofrequency ablation is reported to impact osteolytic and mixed osteolytic-osteoblastic lesions, as opposed to MWA, which offers advantages in the treatment of osteosclerotic lesions [10]. In our case, we preferred not to apply either method of ablation, as we were not sure of the stability we would achieve.

Major spinal surgery is to be considered when there is instability and symptomatic epidural spinal cord compression. However, one should be realistic about the condition of the patient and their overall oncological status; the postoperative complications that may arise after corpectomy or decompression with or without stabilization may include (and are not limited to) dural tears, epidural hematoma formation, dysregulation in blood coagulation, wound healing issues, infection, and prolonged bedrest-associated pneumonia [6,11-12]. Oncological patients are oftentimes in an already poor condition, which does not warrant the excessive perioperative risks. Our patient was relatively healthy, aside from the chronic lymphedema, but she had more than three levels to operate on and was obese; this is an additional factor for poor wound healing if we decided on decompression and open surgery in general.

Bone metastasis results from the mutual selection and interactions between metastatic tumor cells and the bone microenvironment, a concept consistent with the “seed and soil” hypothesis proposed by Paget in 1889. The majority of the breast cancer patient population mainly exhibits osteolytic bone metastases due to enhanced osteoclast activity [13]. Thus, there are more pathologic fractures in these kinds of metastases compared to other oncological conditions, such as prostate cancer. In that line of thinking, there should be more advancement in the less invasive techniques of stabilization of the spinal column so we can better ameliorate the person's quality of life with a lesser extent of surgical intervention.

Breast cancer is one of the most common causes of spinal metastases. In terms of metastatic potential according to histological differences, invasive lobular carcinoma (ILC) is more likely to have bone metastasis than invasive ductal carcinoma (IDC). There are also differences in metastatic potential of breast carcinoma according to the hormonal and HER-2-status with predilection: HER2 status showed that within HER2-positive breast cancers, ER-positive/PR-positive ones still manifest better survival than the other three HR status subgroups, which are similar in survival outcomes [14].

## Conclusions

When considering the optimal patient management approach, the patient’s quality of life must not be underestimated. Open surgery with decompression and fixation of the segment may allow the prevention of segmental instability; however, it is hardly applicable for polymetastatic disease of multiple adjacent levels that have compromised bone structure. The short life expectancy that is predicted for patients who present with multiple spinal metastases does not warrant the increased risks of prolonged hospital stay and postoperative complications that may occur after major spinal surgery. Hence, we believe that percutaneous vertebroplasty to prevent spinal fractures and alleviate pain combined with subsequent radiotherapy is beneficial to patients with multiple spinal metastases.
